# Correction: Effects of Silver Nitrate and Silver Nanoparticles on a Planktonic Community: General Trends after Short-Term Exposure

**DOI:** 10.1371/journal.pone.0107092

**Published:** 2014-08-25

**Authors:** The PLOS ONE Staff

## Notice of Republication

This article was republished on August 11, 2014, to replace an incorrectly changed character in the byline. The publisher apologizes for the error. Please download this article again to view the correct version. The originally published, uncorrected article and the republished, corrected article are provided here for reference.

## Supporting Information

File S1 Originally published, uncorrected article.
(PDF)

File S2 Republished, corrected article.
(PDF)
