# Supplementary material for: Effects of Silver Nitrate and Silver Nanoparticles on a Planktonic Community: General Trends after Short-Term Exposure
Source: PLoS One. 2014 Apr 22;9(4):e95340. doi: 10.1371/journal.pone.0095340 (PMC3995725; doi:10.1371/journal.pone.0095340)
Supplement: Figure S1 — Results of the pre-test experiments. In a preliminary set of experiments we exposed a plankton community from a eutrophic pond at the campus Essen of the University Duisburg-Essen, Germany, to different concentrations of AgNO3, as a basis for selection of silver concentrations to be used in the main experiment. For the pre-tests, approximately 30 mL of pond water were transferred into cell culture flasks and exposed to AgNO3 (0 µg/L, 0.01 µg/L, 0.1 µg/L, 1 µg/L, 10 µg/L, 100 µg/L) under the same experimental settings as described for the main experiment. After 24 h sub-samples were taken from the cell culture flasks and checked for the occurrence of living cells under the light microscope. Since most dead protist cells lyse within minutes to hours, only the living cells were counted. The pretest focused on the abundance of heterotrophic protists. In addition we used one phototrophic dinoflagellate, i.e. Ceratium sp.: Ceratium sp. seemed to be a sensitive indicator organism when exposed to silver. Further, for this species the enumeration of living and dead cells was possible. Therefore, the ratio of living to dead Ceratium sp. individuals were counted as well. Analysis of survival of heterotrophic protists as well as of the ratio living : dead Ceratium sp. showed EC50-values ranging between 1 to 10 µg/L. Accordingly, we have decided to apply a Ag concentration of 5 µg/L. (DOCX) [file pone.0095340.s001.docx]

**Supplementary material**

**Figure S1: Results of the pre-test experiments**

In a preliminary set of experiments we exposed a plankton community from a eutrophic pond at the campus Essen of the University Duisburg-Essen, Germany, to different concentrations of AgNO_3_, as a basis for selection of silver concentrations to be used in the main experiment. For the pre-tests, approximately 30 mL of pond water were transferred into cell culture flasks and exposed to AgNO_3_ (0 µg/L, 0.01 µg/L, 0.1µg/L, 1 µg/L, 10 µg/L, 100 µg/L) under the same experimental settings as described for the main experiment. After 24h sub-samples were taken from the cell culture flasks and checked for the occurrence of living cells under the light microscope. Since most dead protist cells lyse within minutes to hours, only the living cells were counted. The pretest focused on the abundance of heterotrophic protists. In addition we used one phototrophic dinoflagellate, i.e. *Ceratium* sp.: *Ceratium* sp. seemed to be a sensitive indicator organism when exposed to silver. Further, for this species the enumeration of living and dead cells was possible. Therefore, the ratio of living to dead *Ceratium* sp. individuals were counted as well. Analysis of survival of heterotrophic protists as well as of the ratio living : dead *Ceratium* sp. showed EC_50_-values ranging between 1 to 10 µg/L. Accordingly, we have decided to apply a Ag concentration of 5 µg/L.
