# Supplementary material for: Effects of Silver Nitrate and Silver Nanoparticles on a Planktonic Community: General Trends after Short-Term Exposure
Source: PLoS One. 2014 Apr 22;9(4):e95340. doi: 10.1371/journal.pone.0095340 (PMC3995725; doi:10.1371/journal.pone.0095340)
Supplement: Table S1 — Microscopically cell counts of planktonic organisms. (DOCX) [file pone.0095340.s002.docx]

**Supplementary material**

**Table S1: Microscopically cell counts of planktonic organisms**

|  | **(cells/ml)** | **start (repl. 1)** | **(repl.2)** | **(repl.3)** | **control (repl.1)** | **(repl.2)** | **(repl.3)** | **AgNO3 (repl.1)** | **(repl.2)** | **(repl.3)** | **AgNP (repl.1)** | **(repl.2)** | **(repl.3)** |
| --- | --- | --- | --- | --- | --- | --- | --- | --- | --- | --- | --- | --- | --- |
| **Amoebozoa** |  | **0,00** | **0,00** | **0,00** | **0,00** | **2,86** | **0,00** | **0,00** | **0,00** | **0,00** | **0,00** | **0,00** | **0,00** |
| (heterotrophic) | Amoeba | 0,00 | 0,00 | 0,00 | 0,00 | 2,86 | 0,00 | 0,00 | 0,00 | 0,00 | 0,00 | 0,00 | 0,00 |
| **Bacilariophyta** |  | **533,33** | **540,00** | **410,00** | **372,50** | **371,43** | **430,00** | **633,33** | **488,57** | **596,67** | **471,04** | **653,33** | **565,71** |
| (phototrophic) | Amphora sp. | 73,33 | 93,33 | 63,33 | 2,50 | 8,57 | 23,33 | 10,00 | 8,57 | 10,00 | 27,03 | 20,00 | 25,71 |
|  | Asterionella sp. | 80,00 | 40,00 | 90,00 | 67,50 | 65,71 | 83,33 | 146,67 | 45,71 | 43,33 | 30,89 | 66,67 | 45,71 |
|  | Cyclotella sp. | 310,00 | 316,67 | 210,00 | 202,50 | 191,43 | 223,33 | 350,00 | 322,86 | 323,33 | 247,10 | 340,00 | 365,71 |
|  | Melosira sp. | 23,33 | 40,00 | 20,00 | 52,50 | 48,57 | 33,33 | 40,00 | 54,29 | 133,33 | 54,05 | 80,00 | 57,14 |
|  | Nitschia sp. | 46,67 | 50,00 | 26,67 | 47,50 | 57,14 | 66,67 | 86,67 | 57,14 | 86,67 | 111,97 | 146,67 | 71,43 |
| **Charophyta** |  | **73,33** | **120,00** | **40,00** | **57,50** | **48,57** | **106,67** | **73,33** | **102,86** | **136,67** | **104,25** | **173,33** | **120,00** |
| (phototrophic) | Staurastrum sp. | 73,33 | 120,00 | 40,00 | 57,50 | 48,57 | 106,67 | 73,33 | 102,86 | 136,67 | 104,25 | 173,33 | 120,00 |
| **Chlorophyta** |  | **36895,00** | **40404,17** | **42580,83** | **32467,50** | **34590,00** | **37722,50** | **48976,67** | **46650,71** | **44481,67** | **52825,82** | **47097,50** | **51334,29** |
| (phototrophic) | Scenedesmus/ Desmodesmus | 13575,00 | 13087,50 | 12737,50 | 9005,00 | 7750,00 | 13137,50 | 13100,00 | 12630,71 | 12178,33 | 14137,50 | 13444,17 | 14650,00 |
|  | Pediastrum/ Sorastrum | 21833,33 | 26016,67 | 28283,33 | 22350,00 | 26100,00 | 23725,00 | 34700,00 | 32500,00 | 31566,67 | 37225,00 | 31250,00 | 35750,00 |
|  | Pandorina sp. | 853,33 | 840,00 | 1180,00 | 735,00 | 428,57 | 466,67 | 726,67 | 937,14 | 170,00 | 814,67 | 1593,33 | 500,00 |
|  | Coelastrum sp. | 276,67 | 180,00 | 66,67 | 112,50 | 102,86 | 106,67 | 13,33 | 142,86 | 120,00 | 239,38 | 236,67 | 114,29 |
|  | Oocystaceae | 80,00 | 40,00 | 40,00 | 35,00 | 11,43 | 80,00 | 53,33 | 97,14 | 106,67 | 61,78 | 146,67 | 45,71 |
|  | Pteromonas sp. | 210,00 | 180,00 | 193,33 | 130,00 | 117,14 | 166,67 | 183,33 | 160,00 | 180,00 | 146,72 | 176,67 | 205,71 |
|  | Tetratsrum sp. | 66,67 | 40,00 | 80,00 | 100,00 | 80,00 | 40,00 | 200,00 | 182,86 | 160,00 | 200,77 | 250,00 | 68,57 |
|  | Ankistrodesmus sp. | 0,00 | 20,00 | 0,00 | 0,00 | 0,00 | 0,00 | 0,00 | 0,00 | 0,00 | 0,00 | 0,00 | 0,00 |
| **Ciliophora** |  | **68,00** | **136,00** | **90,00** | **96,33** | **90,29** | **111,67** | **106,00** | **83,10** | **115,67** | **186,08** | **152,33** | **90,29** |
| (heterotrophic) | undetermined ciliat 1 | 53,33 | 116,67 | 73,33 | 65,00 | 74,29 | 93,33 | 80,00 | 68,57 | 96,67 | 142,86 | 133,33 | 74,29 |
|  | undetermined ciliat 2 | 0,00 | 6,67 | 6,67 | 20,00 | 0,00 | 3,33 | 10,00 | 2,86 | 6,67 | 30,89 | 3,33 | 0,00 |
|  | Vorticella sp. | 14,67 | 12,67 | 10,00 | 11,33 | 16,00 | 15,00 | 16,00 | 11,67 | 12,33 | 12,33 | 15,67 | 16,00 |
| **Crustacea** |  | **0,00** | **0,00** | **0,00** | **0,00** | **0,00** | **3,33** | **0,00** | **2,86** | **0,00** | **0,00** | **0,00** | **2,86** |
| (heterotrophic) | Bosnia sp. | 0,00 | 0,00 | 0,00 | 0,00 | 0,00 | 3,33 | 0,00 | 2,86 | 0,00 | 0,00 | 0,00 | 2,86 |
| **Cryptophyta** |  | **80,00** | **43,33** | **56,67** | **52,50** | **42,86** | **30,00** | **20,00** | **5,71** | **10,00** | **84,94** | **80,00** | **34,29** |
| (phototrophic) | Cryptomonas sp. | 80,00 | 43,33 | 56,67 | 52,50 | 42,86 | 30,00 | 20,00 | 5,71 | 10,00 | 84,94 | 80,00 | 34,29 |
| **Cyanobacteria** |  | **13800,00** | **17300,00** | **14100,00** | **16625,00** | **12375,00** | **18266,67** | **17850,00** | **18425,00** | **15833,33** | **17433,33** | **19100,00** | **16150,00** |
| (phototrophic) | Microcystis wesenbergii | 13800,00 | 17300,00 | 14100,00 | 16625,00 | 12375,00 | 18266,67 | 17850,00 | 18425,00 | 15833,33 | 17433,33 | 19100,00 | 16150,00 |
| **Dinophyta** |  | **300,00** | **326,67** | **296,67** | **170,00** | **180,00** | **236,67** | **233,33** | **200,00** | **175,00** | **277,99** | **310,00** | **308,57** |
| (mixotrophic) | Gymnodinium sp. | 163,33 | 206,67 | 163,33 | 125,00 | 122,86 | 180,00 | 213,33 | 188,57 | 170,00 | 173,75 | 246,67 | 214,29 |
| (phototrophic) | Ceratium sp. | 136,67 | 120,00 | 133,33 | 45,00 | 57,14 | 56,67 | 20,00 | 11,43 | 5,00 | 104,25 | 63,33 | 94,29 |
| **Euglenophyta** |  | **60,00** | **53,33** | **106,67** | **55,00** | **71,43** | **70,00** | **43,33** | **48,57** | **53,33** | **61,78** | **30,00** | **37,14** |
| (phototrophic) | Euglena sp. | 6,67 | 3,33 | 16,67 | 12,50 | 2,86 | 6,67 | 6,67 | 11,43 | 6,67 | 7,72 | 10,00 | 2,86 |
|  | Monomorphina sp. | 3,33 | 0,00 | 13,33 | 2,50 | 2,86 | 3,33 | 10,00 | 5,71 | 10,00 | 3,86 | 3,33 | 2,86 |
|  | Phacus sp. | 0,00 | 0,00 | 3,33 | 2,50 | 5,71 | 0,00 | 6,67 | 0,00 | 3,33 | 23,17 | 10,00 | 0,00 |
|  | Trachelomonas | 50,00 | 50,00 | 73,33 | 37,50 | 60,00 | 60,00 | 20,00 | 31,43 | 33,33 | 27,03 | 6,67 | 31,43 |
| **Rotifera** |  | **4,00** | **3,33** | **4,33** | **7,00** | **4,67** | **4,67** | **4,33** | **7,86** | **3,33** | **4,67** | **6,00** | **4,67** |
| (heterotrophic) | Keratella sp. | 4,00 | 3,33 | 4,33 | 7,00 | 4,67 | 4,67 | 4,33 | 5,00 | 3,33 | 4,67 | 6,00 | 4,67 |
|  | Undetermined Rotifera | 0,00 | 0,00 | 0,00 | 0,00 | 0,00 | 0,00 | 0,00 | 2,86 | 0,00 | 0,00 | 0,00 | 0,00 |
| **heterotrophic Flagellates** |  | **2985,06** | **2327,85** | **2592,37** | **2878,18** | **2748,15** | **2715,82** | **2078,43** | **3353,38** | **2008,18** | **2418,78** | **3089,63** | **2463,95** |
|  | heterotrophic Flagellates | 2985,06 | 2327,85 | 2592,37 | 2878,18 | 2748,15 | 2715,82 | 2078,43 | 3353,38 | 2008,18 | 2418,78 | 3089,63 | 2463,95 |
| **Sum** |  | **54798,73** | **61254,68** | **60277,54** | **52781,51** | **50525,25** | **59697,99** | **70018,77** | **69368,62** | **63413,84** | **73868,68** | **70692,13** | **71111,76** |
