# Supplementary material for: Correction: Effects of Silver Nitrate and Silver Nanoparticles on a Planktonic Community: General Trends after Short-Term Exposure
Source: PLoS One. 2014 Aug 25;9(8):e107092. doi: 10.1371/journal.pone.0107092 (PMC4143366; doi:10.1371/journal.pone.0107092)
Supplement: File S2 — Republished, corrected article. (PDF) [file pone.0107092.s002.pdf]

PLOS ONE

Effects of Silver Nitrate and Silver Nanoparticles on a Planktonic Community: General Trends after Short-Term Exposure

Jens Boenigk[1]*, Daniela Beisser[2], Sonja Zimmermann[3], Christina Bock[1], Jurij Jakobi[4], Daniel Grabner[3], Lars Großmann[1], Sven Rahmann[2], Stephan Barcikowski[4], Bernd Sures[3]

1 Biodiversity and Centre for Water and Environmental Research, University of Duisburg-Essen, Essen, Germany, **2** Genome Informatics, Institute of Human Genetics, Faculty of Medicine, University of Duisburg-Essen, Essen, Germany, **3** Aquatic Ecology and Centre for Water and Environmental Research, University of Duisburg-Essen, Essen, Germany, **4** Technical Chemistry I, University of Duisburg-Essen and Center for Nanointegration Duisburg-Essen (CENIDE), Essen, Germany

Abstract

Among metal pollutants silver ions are one of the most toxic forms, and have thus been assigned to the highest toxicity class. Its toxicity to a wide range of microorganisms combined with its low toxicity to humans lead to the development of a wealth of silver-based products in many bactericidal applications accounting to more than 1000 nano-technology-based consumer products. Accordingly, silver is a widely distributed metal in the environment originating from its different forms of application as metal, salt and nanoparticle. A realistic assessment of silver nanoparticle toxicity in natural waters is, however, problematic and needs to be linked to experimental approaches. Here we apply metatranscriptome sequencing allowing for elucidating reactions of whole communities present in a water sample to stressors. We compared the toxicity of ionic silver and ligand-free silver nanoparticles by short term exposure on a natural community of aquatic microorganisms. We analyzed the effects of the treatments on metabolic pathways and species composition on the eukaryote metatranscriptome level in order to describe immediate molecular responses of organisms using a community approach. We found significant differences between the samples treated with 5 µg/L $AgNO_3$ compared to the controls, but no significant differences in the samples treated with AgNP compared to the control samples. Statistical analysis yielded 126 genes (KO-IDs) with significant differential expression with a false discovery rate (FDR) <0.05 between the control (KO) and $AgNO_3$ (NO3) groups. A KEGG pathway enrichment analysis showed significant results with a FDR below 0.05 for pathways related to photosynthesis. Our study therefore supports the view that ionic silver rather than silver nanoparticles are responsible for silver toxicity. Nevertheless, our results highlight the strength of metatranscriptome approaches for assessing metal toxicity on aquatic communities.

Citation: Boenigk J, Beisser D, Zimmermann S, Bock C, Jakobi J, et al. (2014) Effects of Silver Nitrate and Silver Nanoparticles on a Planktonic Community: General Trends after Short-Term Exposure. PLoS ONE 9(4): e95340. doi:10.1371/journal.pone.0095340

Editor: Vipul Bansal, RMIT University, Australia

Received November 29, 2013; **Accepted** March 25, 2014; **Published** April 22, 2014

Copyright: © 2014 Boenigk et al. This is an open-access article distributed under the terms of the Creative Commons Attribution License, which permits unrestricted use, distribution, and reproduction in any medium, provided the original author and source are credited.

Funding: Funding of the study by the Centre for Water and Environmental Research (ZWU), University of Duisburg-Essen and DFG project BO3245/3-1 is gratefully acknowledged. The funders had no role in study design, data collection and analysis, decision to publish, or preparation of the manuscript.

Competing Interests: The authors have declared that no competing interests exist.

* E-mail: Jens.Boenigk@uni-due.de

Introduction

Engineered silver nanoparticles (AgNP) are used in a wide variety of applications, for example as antimicrobial additives in textiles, as household products and in medical applications. The recent upward trend in production (estimated 500 t/a worldwide) [1] and application resulted in an increasing release of AgNP as well as of ionic silver into the environment as can be seen from elevated levels of Ag in the aquatic environment [2–5]. So far, the impact of AgNP, as well as of ionic silver species on aquatic organisms has been studied mostly in laboratory experiments using single test species, sometimes even clonal cultures (e.g. *Chlamydomonas* sp.) [6–9]. As a general trend it appears that toxicity of silver is due to ionic silver as the molecular toxicant [10,11]. Nevertheless, toxicity of AgNP is still relevant as particles represent a source from which Ag^+ can be formed continuously with subsequent toxic effects [7].

Realistic assessment of nanoparticle toxicity (mediated by their ionic forms) in natural waters is difficult due to the interaction of nanoparticles and ions with other inorganic and organic molecules [2]. Accordingly, it is necessary to transfer laboratory results to field conditions. Also, the use of single species as test organisms as well as analyses of single parameters such as cell numbers or chlorophyll content will be insufficient if community effects and functional diversity of ecosystems are of interest [12]. In this context, a metatranscriptome sequencing approach is able to elucidate reactions of whole communities present in a water sample to stressors like toxic substances [13]. Differential transcription of genes related to various metabolic pathways (e.g. photosynthesis, fatty acid biosynthesis or glycolysis) is not only linked to single organisms, but shows the ecological functionality of certain groups of taxa in a sample [14–16]. Therefore, this method allows detection of possible environmental hazards in a realistic approach, taking into account the species community as a whole.

To the best of our knowledge, no information exists on the effects of silver nitrate ($AgNO_3$) as compared to AgNP on aquatic communities to date. Accordingly, we compared the toxicity of

ionic silver and AgNP by short-term exposure of a natural community of aquatic microorganisms in a laboratory exposure experiment. Since the activity of AgNP is influenced by the ligands, ligand-free nanoparticles are especially suitable for such comparisons [17]. Effects of the treatments on metabolic pathways and species composition were analyzed on the eukaryote metatranscriptome level in order to describe immediate molecular responses of organisms using a community approach.

Materials and Methods

General Experimental Set Up

A one-day exposure experiment was conducted in June 2013 in a climate chamber at 16°C with homogenously distributed artificial day light. The intensity of the light was 60–78 µE m^{-2} s^{-1} with a 16h/8h light-dark-cycle. Approximately 150 L of water containing a natural plankton community from a eutrophic pond at the campus Essen of the University Duisburg-Essen, Germany, were transferred to a 200 L glass tank. The next day, 10 L of pond water from the glass tank were filled to to nine 20 L plastic tanks respectively and aerated by aquarium pumps. The nine tanks were divided into three experimental groups (control, $AgNO_3$ and AgNP) with three replicate tanks each.

Silver exposure was performed using a Ag-standard solution (ICP-Standard Silber, 1g Ag/L, Bernd Kraft GmbH, Duisburg, Germany) for the $AgNO_3$-group and a freshly laser generated silver nanoparticle suspension for the AgNP-group. For each treatment, silver was added to the water resulting at a nominal Ag concentration of 5 µg/L, which was shown to be sublethal in pre-test experiments (see Figure S1). Monitoring of silver concentrations during exposure was performed by Ag analyses of 10 ml water samples taken from each tank 30 min and 24 h after the start of exposure; from the silver exposed groups one additional water sample was drawn after 5 h following exposure start. Half of the water samples were filtered (0.2 µm, cellulose acetate single use filter, MACHEREY-NAGEL GmbH & Co. KG, Düren, Germany) to remove organisms and organic particles. These samples were considered to reflect the concentration of dissolved silver. All water samples were acidified with 10 µl HNO_3 (subboiled from 65% HNO_3, p.a., Bernd Kraft GmbH, Duisburg, Germany) and were analyzed on the same day. The experiment was terminated after 24 h. Before exposure to silver, samples were taken for the metatranscriptomic sequence analysis (2.5 L of water containing the native plankton community) and for determination of water characteristics (1 L) from the 200 L glass tank. After 24 h of exposure the same sample volumes were taken from each treatment group. Additionally, temperature, pH, conductivity and O_2-concentration were measured twice during the exposure period in every tank.

Preparation of AgNP

Silver nanoparticles were generated via laser ablation of a silver target in liquid aqueous medium according to Barcikowski & Compagnini [18] and Zeng et al. [19]. To this end, a silver foil (Goodfellow, 99.99%) was ablated with a Nd:YAG nanosecond pulsed laser (Rofin PowerLine 20E) at $\lambda = 1064$ nm with a repetition rate of 10 kHz and a pulse energy of 0.3 mJ. The ablation process was carried out in a flow-through chamber with a volume of 1.8 ml, while the target was constantly covered with a liquid layer of 5.5 mm [20]. The carrier stream contained sodium phosphate buffer (pH 7) at an ionic strength of 50 µM and was continuously pumped through the ablation chamber using a peristaltic pump (Ismatec ISM321C) at a constant flow rate of 11.5 mL/min. To avoid the inhibition of silver ion release, which was shown by Grade et al. [17], the generation of silver colloid was carried out without additional stabilizer. Characterization of the sample was done by UV-Vis spectroscopy (Thermo Scientific Evaluation 201), recording spectra from 200–900 nm in a quartz cuvette (volume 3.5 ml, path length 10 mm). A significant plasmon resonance of silver nanoparticles (Fig. 1a) was detected via UV-Vis measurement. A narrow surface Plasmon resonance peak at $\lambda = 393$ nm was found, which indicates the formation of small spherical nanoparticles, while no agglomerates (scattering in the NIR regime) were detected [21]. Particle size and particle size distributions were analyzed via analytical disc centrifugation (CPS Instruments Disc Centrifuge DC24000) and TEM (Philips CM12). TEM micrographs confirmed the formation of spherical nanoparticles with mean particle diameters of 6 nm (distribution PDI 0.33, Fig. 1b).

Metal Analyses and Water Chemistry

Metal analyses of the water samples were carried out by electrothermal atomic absorption spectrometry (ET-AAS) using a Perkin-Elmer model 4110ZL atomic absorption spectrometer equipped with a Zeeman effect background correction system (Perkin-Elmer, Massachusetts, USA). Twenty microlitres of the samples were injected without addition of a modifier in a pyrolytic graphite furnace tube with L'vov platform by the autosampler AS 70 and run under optimized operating parameters with pyrolysis at 600°C and atomization at 1700°C. Calibration was performed by matrix adapted calibration using water of the control group

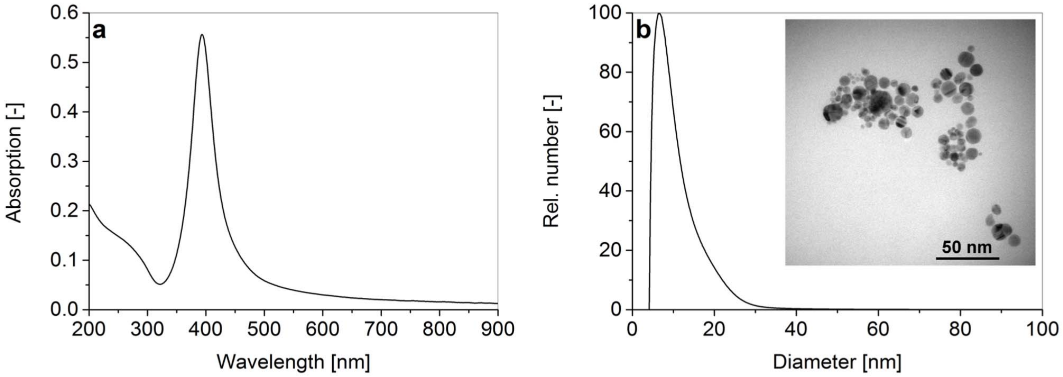

Figure 1. a) Absorption spectrum and b) size distribution data from analytical disc centrifugation of laser-generated silver nanoparticles with an exemplary TEM micrograph (insert).
doi:10.1371/journal.pone.0095340.g001

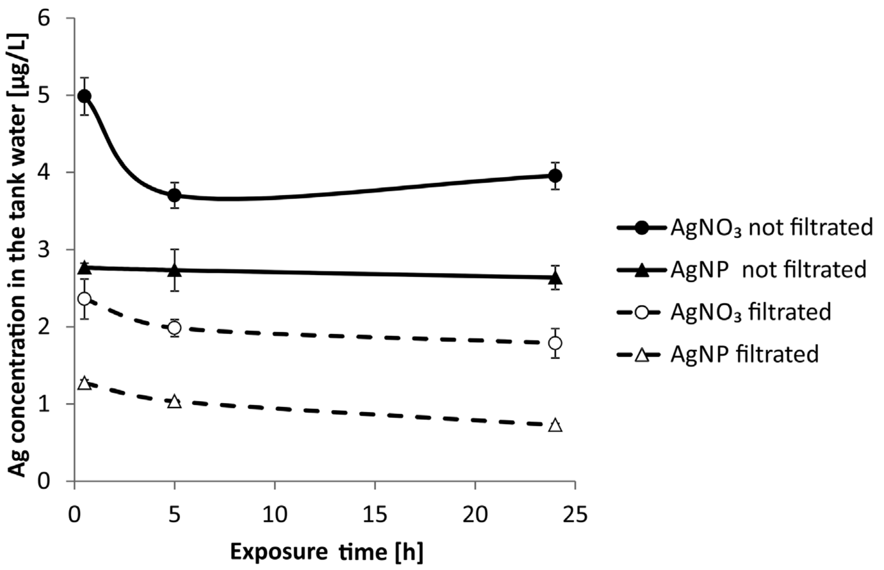

Figure 2. Ag concentrations in the tank water (mean ± SD of three replicate tanks) as determined by ET-AAS. Initially added Ag concentration was 5 µg/L. Ag concentrations in the control were below detection limit.
doi:10.1371/journal.pone.0095340.g002

which was spiked with increasing amounts of Ag. Concentrations of Ag were calculated by fitting linear regression lines to the points defined by the spiked concentration values and the corresponding integrated peak areas in each sample. Correlation coefficients were always >0.99.

The concentration of ammonium, dissolved phosphate, nitrate and nitrite were determined using Spectroquant test kits (Merck KGaA, Darmstadt, Germany). For the determination of the chloride concentration and the carbonate and total hardness titrimetric tests (Merck KGaA, Darmstadt, Germany) were used. The determination of chlorophyll a was performed according to DIN 38412-16:1985-12 [22]. All analyses were performed before and after exposure.

Microscopical Analyses

For the visual analysis of exposure effects, aliquots of the plankton samples were monitored by light microscopy. Therefore, protist communities were analyzed from lugol-fixed samples following established protocols by using Sedgewick Rafter chambers and Utermöhl chambers [23–25]. Protists were analyzed at 200x magnification under an inverted microscope (Nikon Eclipse Ti); for small taxa 400x magnification was applied. Bacteria were counted from formaldehyde-preserved subsamples by means of epifluorescence microscopy (Nikon Eclipse 80i) after DAPI (4',6-diamidino-2-phenylindole) staining at 1000x magnification.

Metatranscriptome Analyses

Following exposure, RNA was extracted from 0.2 µm polycarbonate filters using TRIzol (Life Technologies, Paisley, Scotland - modified). For lysis and homogenization, the cells were ground in liquid nitrogen in a mortar and pestle and incubated for 15 min with TRIzol. RNA, DNA, proteins and lipids were separated in phases by adding chloroform and subsequent centrifugation. The RNA containing aqueous phase was transferred to a clean reaction tube and precipitated with isopropanol. The RNA pellet was washed three times with 75% ethanol and afterwards resuspended in DEPC water. Preparation of the cDNA library as well as sequencing was carried out using an Illumina HiSeq platform via a commercial service (Eurofins MWG GmbH, Ebersberg, Germany). After quality control, one amplified short insert cDNA library (poly-A enriched) with an insert size of 150–400bp was prepared per sample, individually indexed for sequencing on HiSeq 2000 and sequenced using the paired-end module. In the following steps the preprocessing of raw reads was performed and the trimmed and filtered reads were subsequently mapped to the Uniprot database [26] for annotation. Transcript quantification and differential gene expression analysis was conducted thereupon.

Quality Control and Preprocessing of Sequencing Data

The quality control tool FastQC (http://www.bioinformatics.babraham.ac.uk/projects/fastqc/) was used to analyze the quality distribution of the raw reads. Adapter sequences at the ends of the reads were removed using the cutadapt software [27]. Cutadapt was also used to trim bad quality bases with a quality score below 20 and discard reads with a length below 30 bp after trimming. The amount of rRNA in the samples was determined by mapping the reads to the SILVA rRNA database [28] using Bowtie2 [29], a short read aligner that maps sequencing reads efficiently by using a Burrows-Wheeler transformed index. The index was built from the downloaded SILVA database release 111. All quality trimmed reads were mapped as single-end reads against the index to determine the amount of remaining rRNA in each sample. Only the unmapped reads were used for further metatranscriptomic analysis.

Mapping of Metatranscriptome Sequences

All remaining reads were mapped to the UniProt Knowledgebase [26] at the amino acid level using RAPSearch2 [30]. RAPSearch2 uses a reduced amino acid alphabet for a very fast protein similarity search. We built the RAPSearch2 index from the downloaded UniProtKB (version May 2012) and mapped each single read of a read pair against the index. For each pair, the hit with the highest score was chosen as protein annotation. The mapping from UniProt IDs to KEGG Orthology IDs (KO-IDs)

Table 1. Water parameters (mean ± SD).

	Sample date	pH[1]	Temp.[1]	Conductivity[1]	Oxygen[1]	Carbonate hardness[2]	Total hardness[2]	Chloride[2]	Nitrate[2]	Chlorophyll a[2]
			[°C]	[µS/cm]	[mg/L]	[mmol/L]	[mg/L]	[mg/L]	[mg/L]	[µg/L]
Stock	09.07.2013	8.4	17.5	550	9.9	2.5±0.1	103±6	103±1	4.4±1.4	89±8
Control	10.07.2013	8.4±0.1	17.0±0.1	548±4	9.4±0.2	2.4±0.1	105±5	102±3	3.6±1.1	60±3
AgNP	10.07.2013	8.4±0.1	17.2±0.1	549±4	9.3±0.1	2.3±0.1	100±5	100±3	4.5±1.2	62±10
$AgNO_3$	10.07.2013	8.4±0.1	17.0±0.1	548±3	9.2±0.1	2.1±0.1	107±6	101±3	3.0±0.3	68±13

[1]two measurements in three replicate tanks, n = 6; except stock, n = 2).
[2]three replicate tanks, n = 3; except stock, n = 3 of the same tank.
doi:10.1371/journal.pone.0095340.t001

[31] is provided by the UniProt database, and the corresponding KO-IDs were assigned to the reads. Mapping results were summarized as a count matrix of 14100 KO-IDs × 12 samples with the number of counts for each gene (KO-ID) in each sample.

Normalization

The count matrix was normalized using the "weighted trimmed mean of M-values" (TMM) method from the R package EdgeR [32]. This transforms the raw counts into counts per million (CPM) by normalizing for different sample sizes.

Statistical Analysis

To explore sources of variation in the normalized count matrix, we used correspondence analysis (CA), as implemented in the R package vegan [33]. CA is a technique that maps high-dimensional data onto a low-dimensional space (here two-dimensional plot) by singular value decomposition of the correspondence matrix. Each axis reveals relations between groups of samples and data points. Samples and data points having high similarity with respect to this relation have similar coordinates in the plot. For reasons of clarity, only the samples were depicted in the plots.

For testing differential expression of genes between sample groups, the R package EdgeR [32] was used. EdgeR models count data as negative binomial distributed. The gene-wise dispersion is estimated by conditional maximum likelihood, and an empirical Bayes procedure is used to shrink the dispersion by borrowing information between genes. An exact test is used to test for differential expression between groups with a model-based normalization. Generalized linear model (GLM) likelihood ratio tests are used to determine differential expression in complex experiments with multiple factors. The GLM likelihood ratio test was applied to the metatranscriptome count data for each gene to account for all identified sources of variation, yielding a p-value for each KO-ID and treatment group.

To visualize significant differences between groups, the p-values obtained from the statistical tests were plotted as a histogram. If no significant effect is present, the p-values follow a uniform distribution by definition. According to Pounds and Morris [34], a p-value distribution can be modeled by a beta-uniform mixture model, where the signal component is represented by the beta distribution and the null component by the uniform distribution. Thus a beta-uniform mixture model was fitted to the p-value distribution using the R package BioNet [35].

The significantly differentially expressed genes (KO-IDs) from the GLM test were used subsequently in an enrichment analysis. Methods from the R package iSubpathwayMiner [36] and own implementations were used to perform a hypergeometric test for each KEGG pathway. All mappings of genes to KEGG pathways and pathways with a significant enrichment were reported.

Results

Metal Analyses and Water Chemistry

The Ag concentrations in the tank water of the different experimental groups were lower in the filtrated water samples compared to the corresponding unfiltrated samples (Fig. 2). In all tanks the Ag concentrations remained constant after 5 h of exposure, except for the filtrated AgNP samples which showed a slight decrease at the end of the exposure period. No differences were detected for physical and chemical water parameters between the experimental groups and their replicates. Mean ± SD values for each experimental group are summarized in table 1.

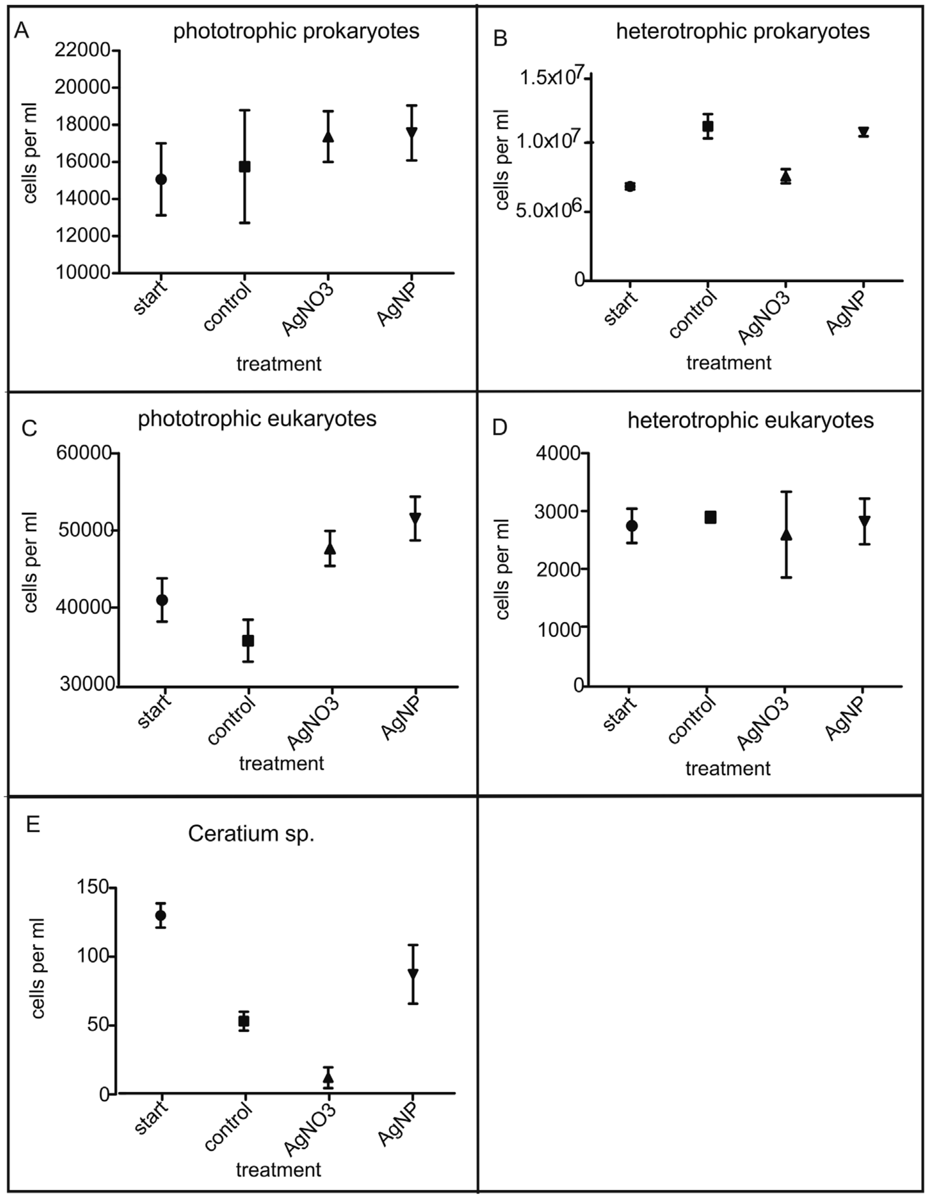

Figure 3. Results of the morphological cell counts: A) phototrophic prokaryotes, B) heterotrophic prokaryotes, C) phototrophic eukaryotes, D) heterotrophic eukaryotes, E) *Ceratium* sp.
doi:10.1371/journal.pone.0095340.g003

Organismic Changes

Microscopic analyses revealed qualitative and quantitative changes within the plankton community (Fig. 3; Table S1). The phototrophic prokaryotes (mainly consisting of *Microcystis wesenbergii*) showed no significant changes between the two silver treatments (Fig.3A). The cell counts of phototrophic prokaryotes were slightly higher in the silver treatments even though this was not significant. Nevertheless, this may indicate a similar effect of silver as observed for the eukaryotic phototrophs (see below). Discrepancies between the two silver exposure groups were documented in the cell counts of heterotrophic prokaryotes. The control and the AgNP treatment nearly had the same mean cell count ($11.2*10^6$ cells/ml and $10.8*10^6$ cells/ml respectively) whereas the mean cell count of $AgNO_3$ was only $7.5*10^6$ cells/ml (Fig. 3B). The dominating phototrophic eukaryotes in all treatments were Chlorophyta, mainly consisting of the groups *Scenedesmus/Desmodesmus* and *Pediastrum/Sorastrum* (see Table S1). Both key groups showed an increase in the mean cell counts in the two silver treatments (Fig. 3C). One taxon which was highly influenced by $AgNO_3$ is *Ceratium* sp. The cell counts showed only 13 individuals in the $AgNO_3$ treatment in comparison to 53 in the control group (Fig. 3E).

Metatranscriptome Sequencing Results

From the 12 environmental RNA samples, between 5.6 and 15.2 million read pairs of 2×100 bp were obtained with good mean quality values of 35. After preprocessing (removal of sequencing adapters, low-quality parts and rRNA reads), we obtained 81% to 92% high-quality reads (Table 2), which were used for the metatranscriptome analysis.

Each remaining read was assigned a UniProt ID and a KEGG Orthology ID (KO-ID). The KO-ID converts the species-specific

Table 2. Summary of sequencing results for each sample.

Sample	Yield (Mbp)	#Reads	%Q30	Mean Q	#Reads R1 trimmed	#Reads R2 trimmed	#Reads mRNA	%HQ mRNA
RAW-1	2,588	12,939,060	90.91	35,06	12,580,021	12,191,810	11,200,104	86.56
RAW-2	2,596	12,982,356	90.76	35,03	12,636,705	12,207,824	11,850,143	91.28
RAW-3	3,039	15,196,013	91.15	35,17	14,984,485	14,462,616	14,047,866	92.44
KO-1	2,541	12,703,316	90.85	35,10	12,279,020	11,915,989	11,642,172	91.65
KO-2	1,986	9,931,991	89.56	34,64	9,508,355	9,105,627	8,729,591	87.89
KO-3	2,087	10,432,882	91.00	35,17	10,101,010	9,785,979	9,566,241	91.69
NO3-1	2,277	11,386,953	91.09	35,16	11,046,068	10,717,279	10,463,186	91.89
NO3-2	2,227	11,136,125	89.81	34,72	10,660,890	10,287,130	9,788,102	87.90
NO3-3	1,873	9,365,969	90.25	34,88	8,914,752	8,654,549	8,440,827	90.12
NP-1	1,813	9,066,148	91.17	35,24	8,900,984	8,559,029	8,313,082	91.69
NP-2	2,911	14,554,562	91.32	35,26	14,229,864	13,764,018	13,174,110	90.52
NP-3	1,125	5,623,772	84.55	32,92	4,818,956	4,660,164	4,565,203	81.18

Summary of the yield in Mbp, the number of raw read pairs, the percentage of reads with a quality value larger than 30 (%Q30), the mean quality value, the number of reads remaining after trimming in forward (R1) and backward (R2) direction, and the number and percentage of remaining high-quality (HQ) read pairs after rRNA removal.
doi:10.1371/journal.pone.0095340.t002

protein annotation from the UniProt database into ortholog groups for all proteins and functional RNAs present in the metatranscriptome samples, independent of the species. This resulted in a gene count matrix of 14100 KO-IDs × 12 samples, where each entry in the table corresponds to the number of times this KO-ID was identified in the sample.

Statistical Analysis Results

We analyzed and compared normalized counts between the four sample groups (raw samples "RAW", control "KO", after treatment with silver nanoparticles "NP", and with silver ions "NO_3") and between the three replicate groups (suffixes "−1", "−2", "−3"). Figure 4 shows a clustering of samples from a correspondence analysis (CA) of the normalized count matrix. On the first two principal axes (CA1, CA2), a clustering due to replicate groups is predominantly visible (Fig. 4A). The four different treatment groups cluster according to the second and third axis (CA2, CA3; Fig. 4B).

After statistical testing for gene-wise differential expression between groups, accounting for the batch effect using a GLM with a multifactor design, we obtain p-values for each gene (KO-ID) and each pair of treatment versus control groups. Figure 5 depicts the obtained p-value distributions. For the test between control and $AgNO_3$ groups (Fig. 5A) we observed a deviation from the uniform p-value distribution induced by the null hypothesis. The deviation emerges from an enrichment of significant p-values outlined by the beta component in a fitted beta-uniform mixture model (BUM model). The beta component of a BUM model describes the signal in the p-value distribution, the amount of significant differences between the two groups, whereas the uniform component arises from the null hypothesis. In contrast, the test between control and AgNP showed no significant results (Fig. 5B). The p-value distribution contained no signal in the NP vs. control tests, as seen by the lack of a beta component in the BUM model. Thus, there are significant differences between the samples treated with $AgNO_3$ compared to the controls, but no significant differences in the samples treated with AgNP compared to the control samples.

Statistical analysis yielded 126 genes (KO-IDs) with significant differential expression with a false discovery rate (FDR) <0.05 between the control (KO) and $AgNO_3$ (NO3) groups. The normalized count matrix for the significant genes is visualized as a bubbleplot in Figure 6, where the counts for each gene are displayed as filled circles. Figure 6A depicts all genes that cannot be annotated to a KEGG pathway, while in Figure 6B up to two pathways are specified for each gene. The 126 significant genes were used for a KEGG pathway enrichment analysis. Three pathways showed significant results with a FDR below 0.05. The enriched pathways were: photosynthesis - antenna proteins, carbon fixation in photosynthetic organisms and photosynthesis (Figure 6 and Table 3).

Data Availability

The raw sequence data in FASTQ format will be made available at the Short Read Archive (SRA) under NCBI accession number SRP040767.

Discussion

In the present study, we analyzed effects of silver applied as $AgNO_3$ and AgNP on the composition and metatranscriptome of a natural community of aquatic protists from a eutrophic pond in a 24 h laboratory exposure. To our knowledge, this is the first approach assessing the impact of silver on ecological functions and

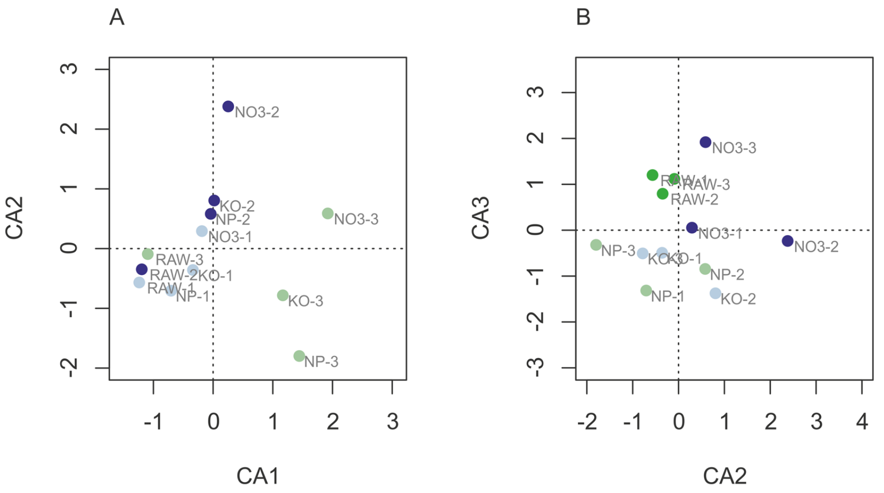

Figure 4. Correspondence analysis of normalized count matrix. Location of samples (RAW, KO, NP, NO3), each in three replicates (−1, −2, −3) is shown after correspondence analysis on the first two principal axes (A: axes CA1, CA2) and on the second and third principal axis (B: axes CA2, CA3). Roughly, the first two axes cluster according to replicate group, while the second and third axes cluster according to treatment.
doi:10.1371/journal.pone.0095340.g004

shifts in species composition on transcriptome level. The silver exposure concentration applied in this experiment was 5 µg/L as either $AgNO_3$ or AgNP. According to pre-trials, higher concentrations were found to alter the planktonic community in a way that was visible microscopically. For example, all eukaryotic organisms were dead after 24 h exposure to a silver concentration of 100 µg/L using $AgNO_3$. Even at a silver concentration of 10 µg/L ($AgNO_3$) fewer flagellates and dinoflagellates survived a one-day exposure compared to unexposed controls. Thus, we opted for a silver concentration of 5 µg/L to guarantee survival of most protist taxa, but similarly to induce effects in the community. This concentration is close to environmentally relevant data which range between 0.01 to 65 µg/L for freshwater ecosystems in Germany [37]. At all concentrations during the pre-trials AgNP exposure resulted in less pronounced effects than ionic silver, which is in line with the assumption that the toxicity and antimicrobial effect of AgNP is considered to be caused by bioavailable Ag^+ ions released from nanoparticles by oxidation [38]. Ion leaching from silver nanoparticles is a process which is influenced by factors such as storage conditions [39], electrochemistry (influence of ions) [40], medium additives (like proteins) [17], and chemicals [41,42]. Simultaneous interactions of these factors, which cannot be analyzed under controlled analytical conditions at once, provide complex kinetics of ion release. In the present study at least the role of biomolecules cannot be excluded completely. This means that not all released ions are bioavailable or toxic. For example, in the case of Ni-alloyed nanoparticles it was shown by Hahn et al. that high ion release from nanoparticles does not correlate with higher toxicity if the ions are bound in complexes by albumin or citrate molecules [43]. Further, it was shown that citrate deactivates silver ion release more than albumin [42]. Since all experiments in the present study were conducted in the same way and we have not registered any effects of the nanoparticles used, no detailed silver leaching experiments from Ag nanoparticles were performed. Generally, toxicity of $AgNO_3$ is higher compared to AgNP [11,3] with toxic concentrations and

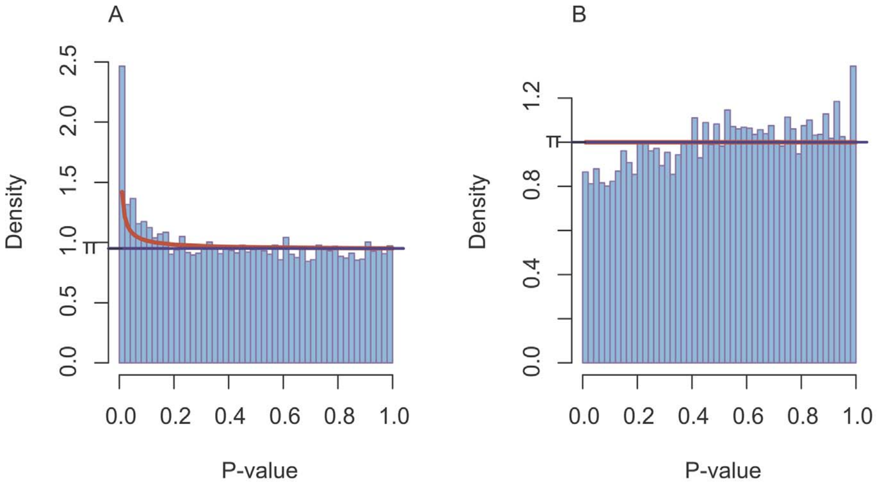

Figure 5. Histogram of p-values after gene-wise tests for differential expression between control vs. $AgNO_3$ (NO3) group (A) and between control vs. silver nanoparticle (NP) group. A beta-uniform mixture model [34] is fitted to the p-value distribution, where the uniform distribution (blue) describes the null component and the beta distribution the enrichment of low p-values (red).
doi:10.1371/journal.pone.0095340.g005

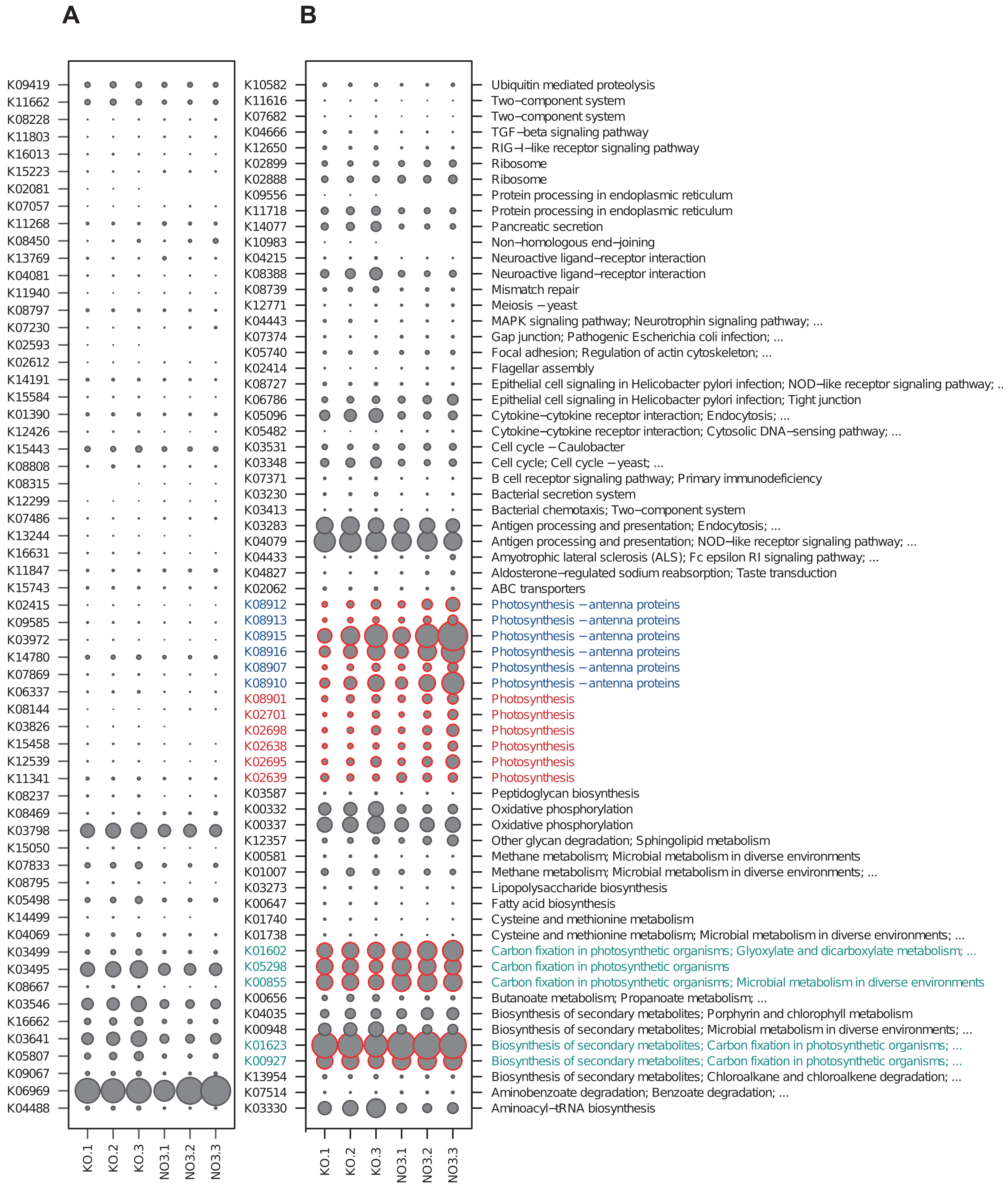

Figure 6. Bubbleplot of count matrix for significant genes with A: genes that do not have an annotated KEGG pathway; B: genes with annotated pathways. Enriched pathways (Table 3) are colored: photosynthesis - antenna proteins: blue, carbon fixation in photosynthetic organisms: turquoise; photosynthesis: red).
doi:10.1371/journal.pone.0095340.g006

inhibitory concentrations being in a range of 0.1 to 20 mg/L Ag^+ for prokaryotes. For eukaryotic cells toxic concentrations of silver ions range between 1 to 10 mg/L and for silver nanoparticles between 10 to 100 mg/L [11]. However, it has to be stressed that only little research has been conducted on toxicity of AgNPs to planktonic taxa [3]. Recently, Ribeiro et al. [44] described

Table 3. Significantly enriched KEGG pathways are shown along with their pathway ID, pathway name, the ratio of genes mapped to the pathway from all selected genes and the ratio of genes belonging to the pathway from all genes in all pathways, p-value for the enrichment and FDR.

pathwayId	pathwayName	annMoleculeRatio	annBgRatio	PValue	FDR
path:00196	Photosynthesis – antenna proteins	6/126	28/14039	1,49E–07	4,46E–05
path:00710	Carbon fixation in photosynthetic organisms	5/126	35/14039	1,41E–05	1,72E–03
path:00195	Photosynthesis	6/126	61/14039	1,72E–05	1,72E–03

doi:10.1371/journal.pone.0095340.t003

impaired reproduction of *Daphnia magna* at 1 µg AgNP/L and 0.5 µg/L (Ag^+) after 21 d of exposure, and feeding rates being affected at 10 µg/L AgNP and 2 µg/L Ag^+ after 24 h exposure. Nevertheless, AgNP can have toxic effects that are higher than expected according to the concentration of dissolved ionic silver [7], probably due to additional effects of particles and agglomerations on cell membranes [45], depending on various factors like media used [46], organic molecules, light conditions and particle size or NP coating [9,47,48]. In the present experiment we detected a toxic effect on protists based on cell counts for the $AgNO_3$ treatments (5 µg/L) and an effect on their gene regulation which is in accordance with the findings of other studies [8]. Effects seem to be taxon-specific, e.g. the presence of a cell wall in a protist species seems to buffer the negative effects of both AgNP and $AgNO_3$ in comparison to those protists lacking one [8,46]. In our study the effects were also clearly species-specific with some taxa such as *Ceratium* being affected stronger than others. However, on the community level we did not observe significant shifts, i.e. the community composition based on higher taxonomic ranks (orders, phyla) remained the same after one-day exposure. This may change following longer exposure periods as it could be shown in a study using a marine mesocosm that bioconcentration and trophic transfer of silver occurred among different taxonomic groups [49].

The AgNP treatments also showed no significant differences in transcriptomic response compared to the control. It may be assumed that only low concentrations of Ag^+ were available to cause effects within the exposure period. On the other hand, the same concentration of $AgNO_3$ showed significant differences in transcriptomic response compared to the control. To avoid a bias in the functional analysis due to the overexpression of a single gene, a misannotation or a false positive significance, we analyzed the changes between the silver treated samples and controls on pathway level. Pathways were reported which were significantly enriched in deregulated genes and therefore the effect of a single gene was compensated. Three pathways were significantly deregulated in the presence of ionic silver: photosynthesis - antenna proteins, carbon fixation in photosynthetic organisms and photosynthesis. The effects of Ag^+ ions on several organisms based on differences in transcribed genes were analyzed in previous studies [15] where photosynthesis was also amongst the most strongly affected pathways. However, genes with photosynthesis-related function were mostly down-regulated after exposure to silver in previous studies [15], but up-regulated in our study. In our experiment all samples were dominated by a bloom of Chlorophytes and no significant change of the Chlorophytes and heterotrophic organisms in the different treatments was observed. In our view, the most likely explanation for the deviating results concerning photosynthesis-related genes are the different concentrations of silver applied in the respective studies. For example, Simon et al. [15] exposed *Chlamydomonas reinhardtii* to a AgNP concentration of 1 mg/L which is 200 times the concentration of our experiment. Silver ions are probably taken up through the cell membrane via the Cu (I) transporter [50], due to similar properties of both metals. Therefore, Cu^+ and Ag^+ might cause a similar cellular response as the cells might not discriminate between the effects of these two metals. Copper ions are known to bind strongly to chlorophyll [51,52] and thus the efficiency of photosynthesis should decrease in the presence of Cu ions. To compensate such an effect, expression of genes relevant for photosynthesis will be up-regulated as we found for $AgNO_3$ in our experiments. Further evidence for such silver effects in the literature are scarce, but Watanabe et al. [53] reported a similarly strong binding of silver to chlorophyll, which may cause an up-regulation of genes related to photosynthesis in the presence of sublethal levels of silver. At higher silver concentrations direct toxic effects on metabolic pathways may be the dominating effects which thus decrease the photosynthesis rate. Such a reduction in photosynthetic yield was reported by Navarro et al. [7] in *Chlamydomonas reinhardtii* exposed to $AgNO_3$ (EC50:188nM after 1 h) and AgNP (EC50:3300M after 1 h) and by Wang et al. [54] in *Raphidocelis subcapitata* exposed to $AgNO_3$ (EC 50:290 nM) and AgNP (EC50:1112.63 µM) after 4.5 h.

Supporting Information

Figure S1 Results of the pre-test experiments. In a preliminary set of experiments we exposed a plankton community from a eutrophic pond at the campus Essen of the University Duisburg-Essen, Germany, to different concentrations of $AgNO_3$, as a basis for selection of silver concentrations to be used in the main experiment. For the pre-tests, approximately 30 mL of pond water were transferred into cell culture flasks and exposed to $AgNO_3$ (0 µg/L, 0.01 µg/L, 0.1 µg/L, 1 µg/L, 10 µg/L, 100 µg/L) under the same experimental settings as described for the main experiment. After 24 h sub-samples were taken from the cell culture flasks and checked for the occurrence of living cells under the light microscope. Since most dead protist cells lyse within minutes to hours, only the living cells were counted. The pretest focused on the abundance of heterotrophic protists. In addition we used one phototrophic dinoflagellate, i.e. *Ceratium* sp.: *Ceratium* sp. seemed to be a sensitive indicator organism when exposed to silver. Further, for this species the enumeration of living and dead cells was possible. Therefore, the ratio of living to dead *Ceratium* sp. individuals were counted as well. Analysis of survival of heterotrophic protists as well as of the ratio living : dead *Ceratium* sp. showed EC_{50}-values ranging between 1 to 10 µg/L. Accordingly, we have decided to apply a Ag concentration of 5 µg/L.
(DOCX)

Table S1 Microscopically cell counts of planktonic organisms.
(DOCX)

Author Contributions

Conceived and designed the experiments: JB SZ CB SB BS SR. Performed the experiments: SZ CB JJ DG LG. Analyzed the data: JB DB SZ CB DG LG SR BS. Contributed reagents/materials/analysis tools: JB SB SR BS. Wrote the paper: JB DB SZ CB JJ DG LG SR SB BS.

References

1. Mueller NC, Nowack B (2008) Exposure modeling of engineered nanoparticles in the environment. Environ Sci Technol 42: 4447–4453.
2. Batley G, Kirby J, McLaughlin M (2012) Fate and risks of nanomaterials in aquatic and terrestrial environments. Accounts Chem 46: 854–862.
3. Fabrega J, Luoma SN, Tyler CR, Galloway TS, Lead JR (2011) Silver nanoparticles: behaviour and effects in the aquatic environment. Environ Int 37: 517–531.
4. Kahru A, Dubourguier H-C (2010) From ecotoxicology to nanoecotoxicology. Toxicology 269: 105–119.
5. Scown TM, van Aerle R, Tyler CR (2010) Review: Do engineered nanoparticles pose a significant threat to the aquatic environment? Crit Rev Toxicol 40: 653–670.
6. Bondarenko O, Juganson K, Ivask A, Kasemets K, Mortimer M, et al. (2013) Toxicity of Ag, CuO and ZnO nanoparticles to selected environmentally relevant test organisms and mammalian cells in vitro: a critical review. Arch Toxicol 87: 1181–1200.
7. Navarro E, Piccapietra F, Wagner B, Marconi F, Kaegi R, et al. (2008) Toxicity of silver nanoparticles to *Chlamydomonas reinhardtii*. Environ Sci Technol 42, 8959–8964.
8. Piccapietra F, Allué CG, Sigg L, Behra R (2012) Intracellular Silver Accumulation in *Chlamydomonas reinhardtii* upon exposure to carbonate coated silver nanoparticles and silver nitrate. Environ Sci Technol 46: 7390–7397.
9. Shi JP, Ma CY, Xu B, Zhang HW, Yu CP (2012) Effect of light on toxicity of nanosilver to *Tetrahymena pyriformis*. Environ Toxicol Chem 31: 1630–1638.
10. Xiu ZM, Zhang QB, Puppala HL, Colvin VL, Alvarez PJJ (2012) Negligible particle-specific antibacterial activity of silver. Nano Lett 12: 4271–4275.
11. Chernousova S, Epple M (2013) Silver as antibacterial agent: ion, nanoparticle, and metal. Angew Chem Int Ed Engl 52: 1636–1653.
12. Cadotte MW, Carscadden K, Mirotchnick N (2011) Beyond species: functional diversity and the maintenance of ecological processes and services. J Appl Ecol 48: 1079–1087.
13. Chen M, Zhang M, Borlak J, Tong W (2012) A decade of toxicogenomic research and its contribution to toxicological science. Toxicol Sci 130: 217–228.
14. Osborn HL, Hook SE (2012) Using transcriptomic profiles in the diatom *Phaeodactylum tricornutum* to identify and prioritize stressors. Aquat Toxicol 138–139: 12–25.
15. Simon DF, Domingos RF, Hauser C, Hutchins CM, Zerges W, et al. (2013) Transcriptome sequencing (RNA-seq) analysis of the effects of metal nanoparticle exposure on the transcriptome of *Chlamydomonas reinhardtii*. Appl Environ Microbiol 79: 4774–4785.
16. Wang Z, Gerstein M, Snyder M (2009) RNA-seq: a revolutionary tool for transcriptomics. Nat Rev Genet 10: 57–63.
17. Grade S, Eberhard J, Neumeister A, Wagener P, Winkel A, et al. (2012) Serum albumin reduces the antibacterial and cytotoxic effects of hydrogel-embedded colloidal silver nanoparticles. RSC Advances 2: 7190–7196.
18. Barcikowski S, Compagnini G (2013) Advanced nanoparticle generation and excitation by lasers in liquids. Phys Chem Chem Phys 15, 3022–3026.
19. Zeng H, Du X-W, Sing C S, Kulinich S A, Yang S, He J, Cai W (2012) Nanomaterials via laser ablation/irradiation in liquid: A review. Adv Funct Mater 22: 1333–1353.
20. Rehbock C, Merk V, Gamrad V, Streubel R, Barcikowski S (2013) Size control of laser-fabricated surfactant-free gold nanoparticles with highly diluted electrolytes and their subsequent biocomjugation. Phys Chem Chem Phys 15: 3057–3067.
21. Menéndez-Manjon A, Schwenke A, Steinke T, Meyer M, Giese U, et al. (2013) Ligand-free gold-silver nanoparticle alloy polymer composites generated by picosecond laser ablation in liquid monomer. Appl Phys A 2: 343–350.
22. Deutsches Institut für Normung (1985) German standard methods for the examination of water, waste water and sludge; test methods using water organisms (group L); determination of chlorophyll a in surface water (L 16) (DIN 38412–16: 1985–12). Berlin: Beuth Verlag GmbH.
23. Auinger BM, Pfandl K, Boenigk J (2008) An improved methodology for the identification of protists and microalgae from Lugol-fixed plankton samples: Combining microscopical analysis with single cell PCR. Appl Environ Microbiol 74: 2505–2510.
24. Medinger R, Nolte V, Panday RV, Jost S, Ottenwälder B, et al. (2010) Diversity in a hidden world: potential and limitation of next generation sequencing for surveys of molecular diversity of Eukaryotic microorganisms. Mol Ecol 19: 32–40.
25. Jost S, Medinger R, Boenigk J (2010) Cultivation independent species identification of Dinobryon sp. (Chrysophyceae) by means of multiplex single cell PCR (MSC-PCR). J Phycol 46: 901–906.
26. Magrane M, UniProt Consortium (2011) UniProt Knowledgebase: a hub of integrated protein data. Database 2011, bar009.
27. Martin M (2011) Cutadapt removes adapter sequences from high-throughput sequencing reads. EMBnet.journal 17: 10–12.
28. Quast C, Pruesse E, Yilmaz P, Gerken J, Schweer T, et al. (2013) The SILVA ribosomal RNA gene database project: improved data processing and web-based tools. Nucleic Acids Res 41: D590–D596.
29. Langmead B, Salzberg SL (2012) Fast gapped-read alignment with Bowtie 2. Nat Methods 9: 357–359.
30. Zhao Y. Tang H, Ye Y (2011) RAPSearch2: a fast and memory-efficient protein similarity search tool for next-generation sequencing data. Bioinformatics 28: 125–126.
31. Kanehisa M, Goto S (2000) KEGG: kyoto encyclopedia of genes and genomes. Nucleic Acids Res 28: 27–30.
32. Robinson MD, McCarthy DJ, Smyth GK (2009) edgeR: a Bioconductor package for differential expression analysis of digital gene expression data. Bioinformatics 26: 139–140.
33. Oksanen J, Blanchet FG, Kindt R, Legendre P, Minchin PR, et al. (2013) vegan: Community Ecology Package. at http://CRAN.R-project.org/package = vegan
34. Pounds S, Morris SW (2003) Estimating the occurrence of false positives and false negatives in microarray studies by approximating and partitioning the empirical distribution of p-values. Bioinformatics 19: 1236–1242.
35. Beisser D, Klau GW, Dandekar T, Müller T, Dittrich MT (2010) BioNet: an R-Package for the functional analysis of biological networks. Bioinformatics 26: 1129–1130.
36. Chunquan L (2012) iSubpathwayMiner: The package can implement the graph-based reconstruction and analyses of the KEGG pathways. at http://CRAN.R-project.org/package = iSubpathwayMiner.
37. Hund-Rinke K, Marscheider-Weidemann F, Kemper M (2008) Beurteilung der Gesamtumweltexposition von Silberionen aus Biozid-Produkten. UBA Text 43/08
38. Kumar R, Howdle S, Münstedt H (2005) Polyamide/silver antimicrobials: effect of filler types on the silver ion release. J Biomed Mater Res B Appl Biomater 75: 311–319.
39. Kittler S, Greulich C, Diendorf J, Köller M, Epple M (2010) Toxicity of silver nanoparticles increases during storage because of slow dissolution under release of silver ions. Chem Mater 22: 4548–4554.
40. Hahn A, Günther S, Wagener P, Barcikowski S (2011) Electrochemistry-controlled metal ion release from silicone elastomer nanocomposites through combination of different metal nanoparticles. J. Mater. Chem. 21: 10287–10289
41. Tiedemann D, Taylor U. Rehbock C, Jakobi J, Klein S, et al. (2014) Reprotoxicity of gold, silver, and gold-silver alloy nanoparticles on mammalian gametes. Analyst 139: 931–942
42. Grade S, Eberhard J, Jakobi J, Winkel A, Stiesch M, et al. (2013) Alloying colloidal silver nanoparticles with gold disproportionally controlls antibacterial and toxic effects. Gold Bull, DOI 10.1007/s13404–013–0125–6
43. Hahn A, Fuhlrott J, Loos A, Barcikowski S (2012) Cytotoxicity and ion release of alloy nanoparticles. J Nanopart Res 14: 686
44. Ribeiro F, Gallego-Urrea J, Jurkschat K, Crossley A, Hassellöv M, et al. (2014) Silver nanoparticles and silver nitrate induce high toxicity to *Pseudokirchneriella subcapitata*, *Daphnia magna* and *Danio rerio*. Sci Total Environ 466–467: 232–241.
45. Lapresta-Fernández A, Fernández A, Blasco J (2012) Nanoecotoxicity effects of engineered silver and gold nanoparticles in aquatic organisms. Trends in Analytic Chemistry, 32: 41–59.
46. Oukarroum A, Bras S, Perreault F, Popovic R (2012) Inhibitory effects of silver nanoparticles in two green algae, *Chlorella vulgaris* and *Dunaliella tertiolecta*. Ecotox Environ Safe 78: 80–85.
47. Liu JY, Hurt RH (2010) Ion release kinetics and particle persistence in aqueous nano-silver colloids. Environ Sci Technol 44: 2169–2175.
48. Liu JY, Sonshine DA, Shervani S, Hurt RH (2010) Controlled release of biologically active silver from nanosilver surfaces. Acs Nano 4: 6903–6913.
49. Cleveland D, Long SE, Pennington PL, Cooper E, Fulton MH, et al. (2012) Pilot estuarine mesocosm study on the environmental fate of silver nanomaterials leached from consumer products: Sci Total Environ 421–422: 267–272.
50. Lee DY, Fortin C, Campbell PGC (2005) Contrasting effects of chloride on the toxicity of silver to two green algae *Pseudokirchneriella subcapitata* and *Chlamydomonas reinhardtii*. AquatToxicol 75: 127–135.
51. Küpper H, Setlik I, Spiller M, Küpper FC, Prasil O (2002) Heavy metal-induced inhibition of photosynthesis: Targets of in vivo heavy metal chlorophyll formation. J Phycology, 38: 429–441.
52. Scotter MJ, Castle L, Roberts D (2005) Method developments and HPLC analysis of retail foods and beverages for copper chlorophyll (E141[i]) and chlorophyllin (E141[ii]) food colouring materials. Food Add Contam, 22: 1163–1175.
53. Watanabe T, Kobayashi M (1988) Chlorophylls as functional molecules in photosynthesis. Molecular composition in vivo and physical chemistry in vitro. J Chem Soc Japan (Nipon Kagaku Kaishi) 4: 383–395.
54. Wang Z, Chen J, Li X, Shao J, Peijnenburg WJGM (2012) Aquatic toxicity of nanosilver colloids to different trophic organisms: contributions of particles and free silver ion. Environ Toxicol Chem 31: 2408–2413.
